# Lower serum uric acid level predicts mortality in dialysis patients

**DOI:** 10.1097/MD.0000000000003701

**Published:** 2016-06-14

**Authors:** Eunjin Bae, Hyun-Jeong Cho, Nara Shin, Sun Moon Kim, Seung Hee Yang, Dong Ki Kim, Yong-Lim Kim, Shin-Wook Kang, Chul Woo Yang, Nam Ho Kim, Yon Su Kim, Hajeong Lee

**Affiliations:** a Department of Internal Medicine, Gyeongsang National University Hospital, Changwon; b Department of Internal Medicine, Seoul National University Hospital; c Department of Internal Medicine, Yeolin Medical Foundation, Seoul; d Department of Internal Medicine, Chungbuk National University Hospital, Cheongju; e Kidney Research Institute, Seoul National University, Seoul; f Department of Internal Medicine, Kyungpook National University Hospital, Daegu; g Department of Internal Medicine, Yonsei University College of Medicine; h Department of Internal Medicine, The Catholic University of Korea College of Medicine, Seoul; i Department of Internal Medicine, Chonnam National University Hospital, Gwangju, Korea.

**Keywords:** end-stage renal disease, mortality, time-averaged serum uric acid

## Abstract

We evaluated the impact of serum uric acid (SUA) on mortality in patients with chronic dialysis. A total of 4132 adult patients on dialysis were enrolled prospectively between August 2008 and September 2014. Among them, we included 1738 patients who maintained dialysis for at least 3 months and had available SUA in the database. We categorized the time averaged-SUA (TA-SUA) into 5 groups: <5.5, 5.5–6.4, 6.5–7.4, 7.5–8.4, and ≥8.5 mg/dL. Cox regression analysis was used to calculate the hazard ratio (HR) of all-cause mortality according to SUA group. The mean TA-SUA level was slightly higher in men than in women. Patients with lower TA-SUA level tended to have lower body mass index (BMI), phosphorus, serum albumin level, higher proportion of diabetes mellitus (DM), and higher proportion of malnourishment on the subjective global assessment (SGA). During a median follow-up of 43.9 months, 206 patients died. Patients with the highest SUA had a similar risk to the middle 3 TA-SUA groups, but the lowest TA-SUA group had a significantly elevated HR for mortality. The lowest TA-SUA group was significantly associated with increased all-cause mortality (adjusted HR, 1.720; 95% confidence interval, 1.007–2.937; *P* = 0.047) even after adjusting for demographic, comorbid, nutritional covariables, and medication use that could affect SUA levels. This association was prominent in patients with well nourishment on the SGA, a preserved serum albumin level, a higher BMI, and concomitant DM although these parameters had no significant interaction in the TA-SUA-mortality relationship except DM. In conclusion, a lower TA-SUA level <5.5 mg/dL predicted all-cause mortality in patients with chronic dialysis.

## Abstract 2

Supplemental Digital Content is available in the text

## 1 Introduction

Unlike the majority of mammals, uric acid is the final product of purine nucleotide metabolism in humans because of the loss of uricase activity during evolution.^[1]^ This loss of uricase activity, the uric acid balance in the kidneys, and lifestyle changes with eating habits are associated with a recent high prevalence of hyperuricemia. Hyperuricemia causes uric acid crystals to deposit in the joints and kidneys, and consequently causes gout and chronic interstitial nephritis. Moreover, increasing data suggest that a higher serum uric acid (SUA) level may be a risk factor or biomarker for both renal and cardiovascular outcomes in a range of populations. In the general population, a higher SUA level is linked to the development of new-onset chronic kidney disease (CKD)^[^^2^,^3^,^4^^]^ and end-stage renal disease (ESRD).^[^^5^,^6^^]^ In addition, an elevation of SUA level has been shown to be associated with hypertension,^[7]^ diabetes mellitus (DM), metabolic syndrome, coronary disease,^[8]^ and even cardiovascular mortality.^[^^8^,^9^,^10^^]^

The association between the SUA level and patients’ outcome seems complex in those with kidney disease. In humans, two-third of the uric acid produced is eliminated in the urine. Therefore, the SUA concentration is determined by both the rate of purine metabolism and the efficiency of renal clearance. Consequently, an elevation of the SUA level is highly prevalent in patients with CKD.^[^^11^,^12^^]^ This makes it difficult to determine a causal or coincidental relationship between the SUA level and outcome in the CKD population compared with the general population. Previous studies on the effect of the SUA level on either a decline in renal function or cardiovascular disease have controversial findings.^[^^13^,^14^,^15^^]^ Additionally, higher SUA levels have been reported to be associated with increased^[^^16^,^17^^]^ or neutral effects^[18]^ on mortality in patients with CKD. Regarding the patients with ESRD, the impact of SUA level on their mortality remains more obscure. In patients on hemodialysis (HD), a J-shaped relationship between the SUA level and mortality has been suggested,^[19]^ whereas several studies have demonstrated that hyperuricemia is protective against mortality^[^^20^,^21^^]^ and cardiovascular events.^[22]^ In peritoneal dialysis, a higher SUA level was found to be harmful in residual renal function,^[23]^ endothelial dysfunction,^[24]^ and cardiovascular events and mortality.^[^^25^,^26^^]^ However, most of these previous studies were retrospective, single-center trials with a single measurement from small population size.

In contrast to the common notion that SUA is a cardiovascular risk factor, SUA is one of the most important antioxidants in human biologic fluids.^[27]^ It is thought that SUA contributes to more than half of the antioxidant capacity of blood.^[^^28^,^29^^]^ This antioxidative potential of SUA may be protective in patients with ESRD who have higher oxidative stress than individuals with preserved renal function. Therefore, we hypothesized that a lower SUA level may increase the mortality risk in patients with ESRD. To prove this hypothesis, we explored the association between the SUA level and mortality in patients with ESRD by using a nationwide representative prospective ESRD cohort in Korea. Because SUA may be influenced on various conditions such as dialysis modality, efficiency of dialysis, and nutritional status, we used time-averaged SUA (TA-SUA) to represent SUA level more precisely in dialysis patients.

## 2 Methods

### 2.1 Study population

We enrolled patients with ESRD between August 2008 and September 2014 through an ongoing cohort study (Clinical Research Center for End Stage Renal Disease [CRC for ESRD]) in South Korea. The CRC for ESRD is a nationwide, multicenter, web-based, prospective cohort of patients with CKD who have started dialysis. This investigation was registered as a clinical trial (NCT00931970).^[30]^ CRC for ESRD had enrolled a total of 4132 adult (age ≥18 years) ESRD patients from 31 dialysis centers. Among them, we included patients who maintained dialysis for at least 3 month and had more than 2 SUA levels. We excluded patients who were initiated on dialysis within 3 months or had no available SUA level. All the patients were informed about the study and participated voluntarily after providing written consent. The study was approved by the institutional review boards of each center.^[31]^

### 2.2 Clinical parameters

The clinical data were collected in the form of web-based medical questionnaires (http://webdb.crc-esrd.or.kr). Well-trained data coordinators completed the questionnaire items by reviewing medical records or conducting in-person interviews. Baseline information at the time of enrollment included age, sex, height, weight, dialysis duration, total Kt/V, primary renal disease, and comorbidities. The dialysis duration was defined as the duration of time between the dialysis initiation date and the patient enrollment date, which was calculated in months. To exclude any medication effect on SUA levels, we gathered information about uricosuric and antiuricosuric drugs. For uricosuric agents, losartan and calcium channel blocking antihypertensive medications were included. In addition, we obtained concomitant use of aspirin, angiotensin receptor blockades except losartan, angiotensinogen-converting enzyme inhibitors, and β-blockers and diuretics as antiuricosuric agents.

At the time of enrollment, we checked patients’ systolic and diastolic blood pressure, weight, and height. Body mass index (BMI) was calculated as weight in kg ÷ (height in m).^[2]^ As the SUA level may be substantially affected by one's nutritional status, we focused on collecting data associated with the patients’ nutritional status. First, we evaluated the subjective global assessment (SGA) to determine the patients’ overall protein-energy nutritional status. The SGA includes 6 subjective assessments: 5 based on the patient's history of weight change, dietary intake, gastrointestinal symptoms, functional impairment, and comorbidities as related to his or her nutritional needs; and 1 based on the physical examination of loss of subcutaneous fat, muscle wasting, ankle edema, sacral edema, and ascites. Each assessment was given a score of 1 to 7, indicating well nourished (6–7), mild to moderately malnourished (3–6), and severely malnourished (1–2). Second, we collected laboratory data such as the plasma hemoglobin, serum creatinine, albumin, calcium, phosphorus, and highly sensitive c-reactive protein (hs-CRP) levels. During the follow-up period, SUA levels were collected every 12 months with other laboratory and clinical data. Averaged SUA was obtained for up to 12 months to calculate the TA-SUA. The blood samples were collected prior to dialysis and meal.

### 2.3 Outcome measurement

The primary outcome was all-cause mortality after enrollment. The dates on mortality and cause of death were reported within 1 month after the event. In patients who withdrew from the study, we ascertained the mortality data from Statistics Korea.^[32]^ We combined all these data according to the unique identification number held by all Koreans who provided informed consent. It was assumed that patients who did not have a reported death did not meet the primary outcome at the time of data close-out. In addition, the causes of death were divided into 8 categories: cardiac, vascular, infection, liver disease, gastro-intestinal, metabolic, endocrine causes, and other. The causes of death were collected for 75% of those who died.

### 2.4 Statistical Analysis

To compare the baseline characteristics according to the TA-SUA level, participants were stratified into 5 groups of TA-SUA levels as follows: <5.5, 5.5–6.4, 6.5–7.4, 7.5–8.4 (reference), and ≥8.5 mg/dL. Differences among the TA-SUA groups were tested using the χ^2^ test for categorical variables, and the analysis of variance *t* test for continuous variables. To assess the relationship between the TA-SUA level and demographic and clinical data, univariate and multivariate linear regression analyses were performed. Variables that showed a significant association (*P* <0.10) in univariate analysis (age, sex, the dialysis type, BMI, SGA, albumin level, TA-SUA, and diabetes) or were of considerable theoretical relevance (total Kt/V, calcium level, phosphorus level, hypertension, gout, uricosuric drugs, and antiuricosuric drugs) were entered into the multivariate stepwise linear regression models. Covariables with colinearity were excluded from multivariate analyses.

To explore the association between the TA-SUA level and mortality in patients on dialysis, a Kaplan–Meier curve was plotted according to the 5 TA-SUA groups. Also, we fit a restricted cubic spline function. From these analyses, we restratified participants into 2 groups of TA-SUA levels (TA-SUA <5.5 mg/dL vs. ≥5.5 mg/dL). Survival differences were compared by the log-rank test. To calculate the relative risk of death, hazard ratios (HRs) and 95% confidence intervals (CI) were obtained by Cox proportional hazards models. The proportional hazard assumption for the Cox model was tested by log minus log plots. Factors that showed a significant association (*P* <0.10) after univariate analysis or were of important clinical concern were entered into the time-dependent multivariate Cox regression analysis. Variable selections were performed using a forward conditional method. Since the association between the TA-SUA <5.5 mg/dL and mortality in patients on dialysis may be a feature of malnutrition, we dissected this relationship according to the SGA, albumin level, and BMI. We also performed subgroup analyses according to age because these parameters were proven to affect the SUA level independently. Statistical analyses were performed using SPSS, version 21.0 for Windows (SPSS Inc, Chicago, IL) and STATA, version 12.0 (StataCorp LP, College Station, TX). Statistical significance was defined as a *P* <0.05.

## 3 Results

### 3.1 Comparison of the characteristics according to the time-averaged SUA level

**Figure 1.**
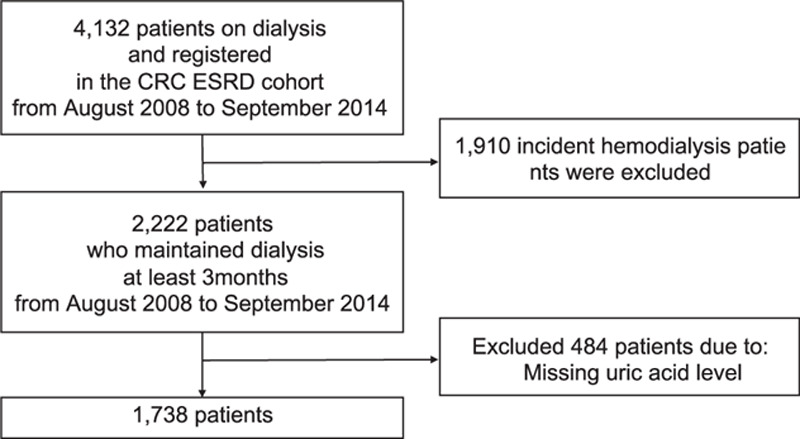
Flow chart of study enrollment. Between August 2008 and September 2014, a total of 1739 prevalent dialysis patients were included.

**Figure 2.**
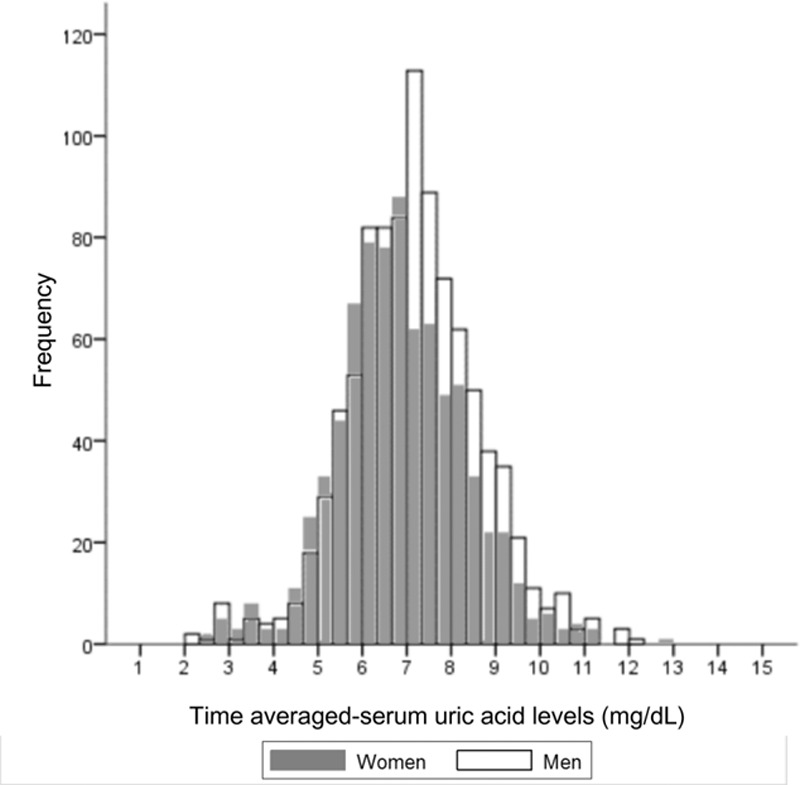
Distribution of the time-averaged serum uric acid levels according to sex. Most of the time-averaged serum uric acid levels in men (white empty bar) overlap with those in women (gray-filled bar), although they are slightly deviated to the right side.

Among a total of 4132 patients in the CRC-ESRD cohort, we excluded 1910 incident hemodialysis patients and 484 patients without available SUA levels. Therefore, 1738 patients were included in the final analysis (Fig. 1). Among them, 45.2% were women (n = 786) and 54.8% were men (n = 952). The mean age was 56 ± 13 years, with a median follow-up of 43.9 months. Fig. 2 shows the TA-SUA level distributions according to sex. The TA-SUA level distribution was not much different between men and women, although the TA-SUA level was slightly higher in men than in women (7.29 ± 2.70 mg/dL vs. 6.88 ± 1.66 mg/dL, *P* <0.001).

Table 1 presents the different baseline characteristics by the TA-SUA level. As the TA-SUA level decreased, the participants were older and more were women. Patients with a lower TA-SUA level tended to have higher proportions of malnourishment (the lowest TA-SUA group, 16.6% vs. the highest TA-SUA group, 7.9%; *P* = 0.019). Additionally, they had a lower BMI, serum phosphorus, triglyceride, low-density lipoprotein cholesterol, total protein, and albumin levels than patients with higher TA-SUA levels. However, dialysis efficiency was better in the lower TA-SUA groups than in the higher TA-SUA groups (*P* < 0.001). Although insignificant, as the TA-SUA level decreased, the proportion of diabetes was increased, whereas that of gout was decreased in comorbid conditions (*P* > 0.05). When we analyzed baseline characteristics divided by dialysis type, most baseline characteristics showed similar trend according to SUA groups in HD and PD patients (Supplementary Table 1, 2).

**Table 1:**
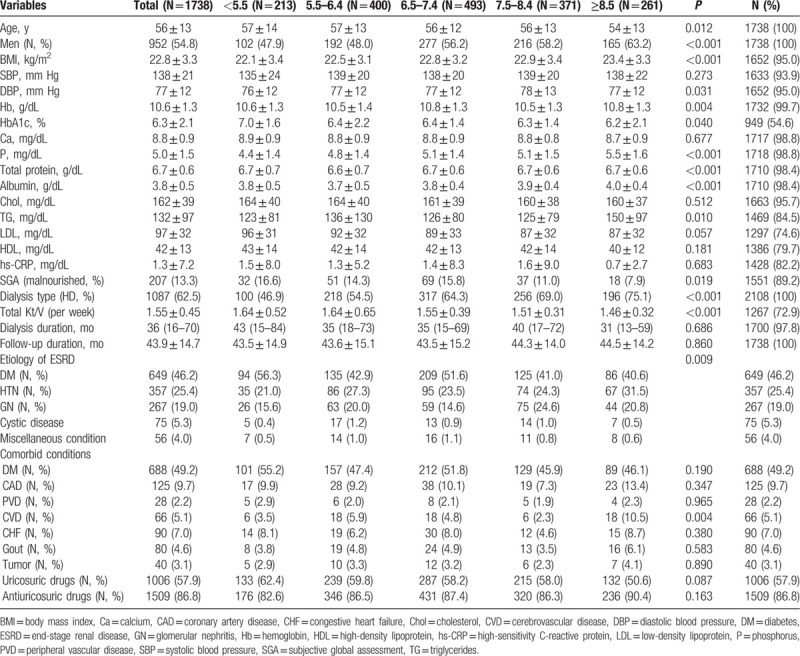
Patient characteristics by the time-averaged serum uric acid groups.

### 3.2 Clinical parameters affecting the time-averaged SUA level

Next, we explored the baseline parameters affecting the TA-SUA level in our population. Table 2 summarizes the results. The TA-SUA level had a significant positive correlation with nutritional factors such as the BMI (regression coefficient [*B*] = 0.042, *P* *=* 0.091), SGA (*B* = −0.062, *P* = 0.014), serum phosphorus level (*B* = 0.107, *P* <0.001), and albumin level (*B* = 0.127, *P* <0.001). The levels of hs-CRP and gout did not show any relationship. Regarding the dialysis type, patients on peritoneal dialysis (PD) were associated with lower TA-SUA levels. Antiuricosuric drugs were significantly associated with the TA-SUA (*B* *=* −0.067*, P* *=* 0.002), while uricosuric drugs were not (*B* *=* 0.014*, P* *=* 0.517). Interestingly, there was an inverse association between the TA-SUA levels and DM (*B* = −0.052, *P* = 0.051). After making an adjustment, we found that PD, a lower BMI, lower albumin levels, lower serum phosphorus levels, and combined DM were significantly associated with lower SUA levels.

**Table 2:**
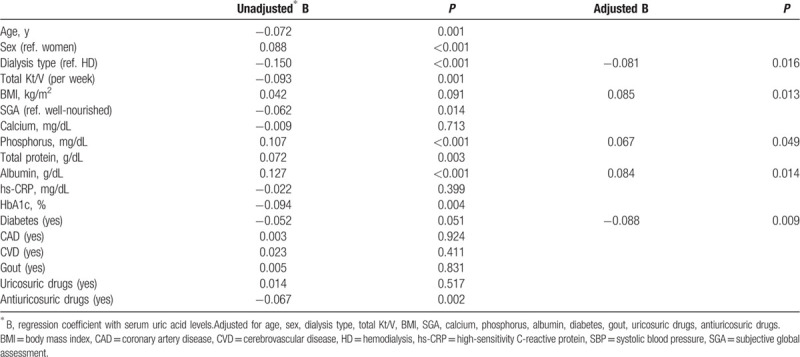
Baseline parameters associated with time-averaged serum uric acid levels.

### 3.3 Survival analyses

**Figure 3.**
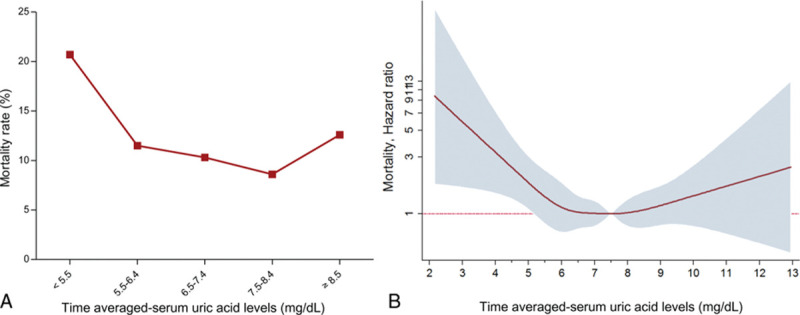
Mortality rates and hazard ratios according to the time-averaged serum uric acid level. This graph shows the crude mortality rate according to the TA-SUA groups. The lowest TA-SUA group has a significantly increased mortality rate compared with the other 4 TA-SUA groups. The log hazard ratios for mortality in relation to the TA-SUA level are presented. A U-shape relationship is plotted between mortality and the TA-SUA level in patients with end-stage renal disease.

During the follow-up periods, 206 of 1738 (11.9%) patients died. The all-cause mortality rate was higher in patients on PD than in those on HD (16.3% [106/651] vs. 9.2% [100/1087], *P* <0.001). Figure 3A illustrates the categorical mortality rate. The highest TA-SUA group had a similar mortality rate to the middle 3 TA-SUA groups. However, the lowest TA-SUA group had a steeply elevated mortality rate compared with the other 4 TA-SUA groups. Figure 3B shows nonlinear mortality risk according to the TA-SUA level after adjusting for clinical covariates such as age, sex, the dialysis type, SBP, BMI, diabetes, albumin level, and SGA. In terms of the nonlinear spline curve, there was an overall U-shape association between the TA-SUA level and adjusted log HR. Different from a wide range of CIs of the mortality risk for higher TA-SUA levels, an increase in the mortality risk was more prominent in the lower TA-SUA levels with a narrow CI range. Moreover, this association was obvious for the TA-SUA level <5.5 mg/dL in terms of both the HR and CI. Subsequently, we performed survival analyses by stratifying the TA-SUA into 2 groups: TA-SUA <5.5 mg/dL and TA-SUA ≥5.5 mg/dL.

**Figure 4.**
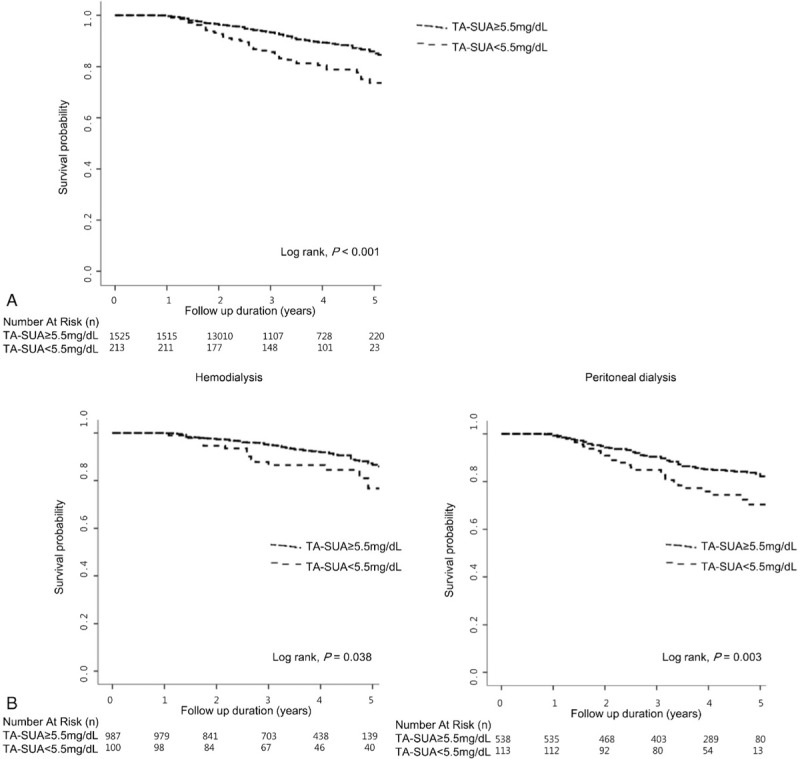
Kaplan–Meier curve according to the time-averaged serum uric acid level above and below 5.5 mg/dL. A, Patients with the TA-SUA <5.5 mg/dL had a higher mortality rate compared with those with TA-SUA ≥5.5 mg/dL. B, A similar trend was observed when divided into HD and PD groups. TA-SUA = time-averaged serum uric acid.

Figure 4 shows the survival curves obtained using the Kaplan–Meier method. Patients with the TA-SUA <5.5 mg/dL had a significantly higher mortality rate compared with those with TA-SUA ≥5.5 mg/dL (log rank, *P* <0.001). A similar trend was observed when divided into HD and PD groups (Fig. 4B). To explore the effect of the TA-SUA level on all-cause mortality, we performed Cox regression analyses. Table 3 summarizes the results of univariate and multivariate analyses. In univariate analysis, we found that the TA-SUA <5.5 mg/dL was significantly associated with all-cause mortality (unadjusted HR, 2.003; 95% CI, 1.435–2.795; *P* <0.001). Moreover, the TA-SUA <5.5 mg/dL was an independent risk factor for all-cause mortality even after adjustment for clinical covariates such as age, dialysis type, total Kt/V, BMI, SGA, serum calcium, phosphorus, albumin, TA-SUA, DM, hypertension, gout, uricosuric drugs, and antiuricosuric drugs (adjusted HR, 1.720; 95% CI, 1.007–2.937; *P* = 0.047).

**Table 3:**
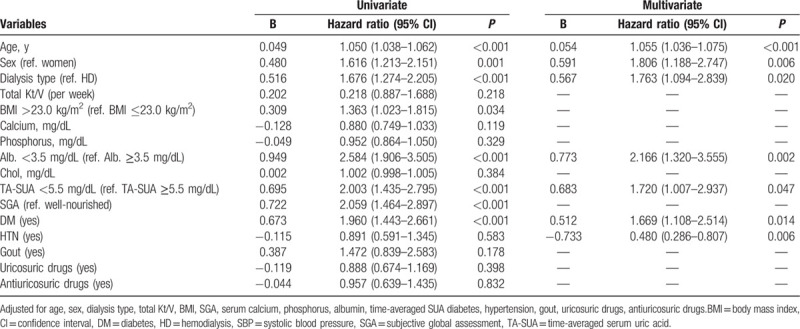
Hazard ratios for mortality risk factors.

### 3.4 Subgroup analyses

**Figure 5.**
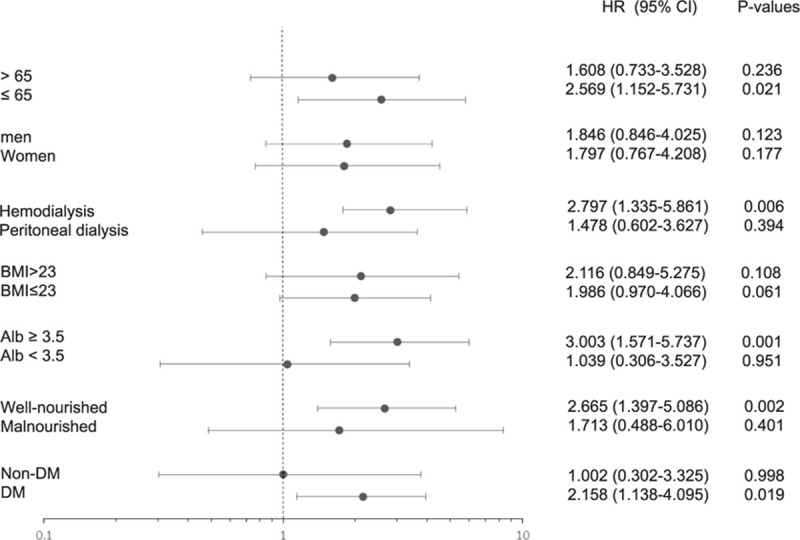
Stratification analyses. A comparison of the adjusted hazard ratios for the subgroups is presented by forest plot. ^a^ Adjusted for age, sex, the dialysis type, body mass index, systolic blood pressure, calcium level, phosphorus level, albumin level, total cholesterol level, uric acid level, subjective global assessment, and DM for each subgroup (excluding its own group).

Since the association between the SUA <5.5 mg/dL and mortality in patients on dialysis may be a feature of malnutrition, we dissected this relationship according to the SGA, albumin level, and BMI. We also performed subgroup analyses according to age, sex, and the dialysis type because these parameters were proven to affect the SUA level independently, as described in Table 2. Figure 5 summarizes the results. There were no significant interactions between the subgroups except DM. The harmful effect of the TA-SUA <5.5 mg/dL level on all-cause mortality was more prominent in age ≦65 years group (adjusted HR, 2.569; 95% CI, 1.152–5.731; *P* = 0.021; *P* for interaction = 0.365), patients with HD treatment (adjusted HR, 2.797; 95% CI, 1.335–5.861; *P* = 0.006; *P* for interaction = 0.746), overweight (BMI >23 kg/m^2^) (adjusted HR 2.116, 95% CI, 0.849–5.275; *P* = 0.108; *P* for interaction = 0.384), normoalbuminemic (adjusted HR 3.003, 95% CI, 1.572–5.737; *P* = 0.001; *P* for interaction = 0.978), well-nourished (adjusted HR 2.665; 95% CI, 1.397–5.086; *P* = 0.002; *P* for interaction = 0.313), and patients with DM (adjusted HR 2.158; 95% CI, 1.138–4.095; *P* = 0.024; *P* for interaction = 0.019). The harmful effect of the TA-SUA was similar between women (adjusted HR, 1.797; 95% CI, 0.767–4.208; *P* = 0.177) and men (adjusted HR, 1.846; 95% CI, 0.846–4.025; *P* = 0.123; *P* for interaction = 0.463).

### 3.5 Causes of deaths

Table 4 shows the causes of death according to the TA-SUA levels. Overall, the 2 most common causes of death were infection (n = 62, 30.1%) and a cardiogenic cause (n = 54, 26.2%), and the distribution of causes of death did not differ according to the TA-SUA levels.

**Table 4:**
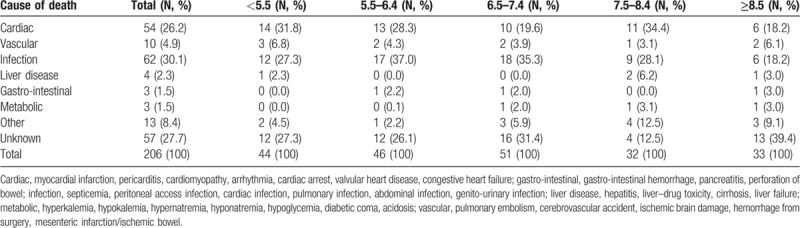
Cause of death according to time-averaged serum uric acid groups.

## 4 Discussion

In this study, we explored the relationship between the TA-SUA level and all-cause mortality in maintenance dialysis patients. SUA level was easily affected by diet, efficiency of dialysis, and drugs. Notably, we used the TA-SUA from the blood samples that were collected prior to dialysis and meal, instead of a single-baseline measurement of SUA. We found that distribution of the TA-SUA level was not much different according to sex as much in the general population. As we expected, the TA-SUA level was associated with patients’ nutritional status. We also demonstrated that a lower TA-SUA level, especially the lowest (<5.5 mg/dL), among our dialysis groups was independently associated with all-cause mortality, over a median follow-up of 43.9 months. This relationship remained significant after considering the nutritional status and any other parameters associated with the TA-SUA levels.

The reference range of SUA is typically 3.4 to 7.2 mg/dL (200–430 μmol/L) for men and 2.4 to 6.1 mg/dL (140–360 μmol/L) for women. Such a sex difference has been well described in previous studies performed in the general population.^[33]^ In patients on dialysis, there are limited data about sex differences of the SUA level distribution. A few previous studies have shown that the SUA level of men is higher than that of women by >0.5 mg/dL.^[^^16^,^34^^]^ In our study, the difference was <0.3 mg/dL, but it was significant. The reason for this difference in our cohort remains uncertain. An advanced age and menopausal status,^[^^35^,^36^^]^ impaired renal uric acid excretion state, and sex hormonal dysfunction in the uremic status^[37]^ may affect the SUA metabolism, although further studies are warranted to clarify this smaller sex difference of the SUA level and its mechanism.

The relationship between the SUA level and mortality in those with ESRD has been explored in 5 HD cohorts^[^^16^,^19^,^20^,^21^,^38^^]^ and 3 PD cohorts,^[^^25^,^26^,^39^^]^ and the results were diverse. Several studies showed that a low SUA level may be harmful against mortality,^[^^16^,^19^,^20^,^21^,^26^^]^ whereas others insisted that hyperuricemia increased mortality.^[^^25^,^38^^]^ One study even reported that the prognostic value of SUA on mortality was weak, and it may be confounded by traditional cardiovascular risk factors or uremia-related factors.^[39]^ This heterogeneity may be derived by many confounders that affect SUA levels in patients on dialysis.

First, SUA can be considered one of the nutritional factors in patients on dialysis. A recent study clarified the association between SUA levels and the nutritional status in patients on HD.^[34]^ Therefore, nutritional markers should be finely adjusted to demonstrate the independent prognostic value of SUA on mortality in patients with ESRD. In our prospective study, we considered all possible nutritional factors, including the SGA, BMI, serum albumin, and phosphorus levels. BMI is a paradoxical variable for mortality in patients with ESRD.^[40]^ Hypoalbuminemia is also well established as a potent independent risk factor for morbidity and mortality in patients on dialysis.^[41]^ Aside from laboratory markers, we also considered the SGA, which represents the overall concepts of nutritional factors such as the subjects’ recent weight change, dietary intake, and gastrointestinal symptoms, loss of subcutaneous fat, and signs of muscle wasting.^[42]^ In patients with ESRD, the SGA was an independent predictor for mortality in a large longitudinal cohort study.^[43]^ In our subgroup analyses, an independent role of the lowest SUA level on elevated mortality was prominent in the well-nourished (on SGA), higher BMI, and higher serum albumin level group than each counterpart. This implies that the SUA level is not a simple marker that reflects the nutritional status of patients with chronic dialysis.

Another important issue to address is that both hyper- and hypouricemia can be associated with an increased mortality. Two previous studies on patients on PD illustrated this common concept by showing that hyperuricemia was associated with an increased all-cause, especially cardiovascular mortality.^[^^25^,^26^^]^ However, 1 study enrolled patients with a relatively young age and fewer comorbidities compared with our cohort.^[26]^ The other study had some limitations, including a small sample size and short follow-up duration.^[25]^ Our study participants were observed prospectively at nationally distributed multicenters, and therefore they are representative of real-world patients with chronic dialysis. Our study demonstrated that a lower TA-SUA level increased the all-cause mortality in patients with chronic dialysis. The SUA is a powerful scavenger for oxygen-free radicals and acts as one of the most important antioxidants in human biological fluids.^[^^27^,^44^^]^ There is enough evidence that a lower SUA level is associated with inflammatory and degenerative diseases.^[^^22^,^29^,^45^,^46^,^47^,^48^^]^ In patients on dialysis, it was found that the total antioxidant capacity was correlated with SUA levels due to primarily higher levels of SUA.^[49]^ Therefore, it is possible that a lower SUA level results in a reduced total antioxidant capacity in patients on dialysis, although further investigations are warranted to clarify the precise mechanisms.

The strengths of our study are as follows: this was a prospective cohort study with a large number of patients treated with PD or HD at multiple centers; we completed a detailed assessment and adjusted for nutritional, metabolic risk factors and drugs, which were the most important confounders of the SUA level; we consider dialysis-related factors that may have eliminated the SUA in patients with limited or absent renal function; we consequently suggested an important but underestimated role of SUA as an antioxidant in patients on dialysis, and we used TA-SUA instead of single SUA measurement to minimize fluctuating effect of SUA.

However, several limitations should be noted. First, we could obtain only partial information about medication that may have changed the SUA levels. Since this study had a prospective cohort design, we did not obtain this information at the time of the study design. Although we could not gather the medication for gout treatment, but we could adjust gout history, uricosuric agents, and antiuricosuric agents. Even after adjusted the gout and drugs affecting SUA levels, the TA-SUA <5.5 mg/dL was an independent risk factor for all-cause mortality. Second, we also could not consider the other antioxidative parameters that may provide one of the relationships between SUA and antioxidant capacity. Among the usual laboratory tests, total bilirubin and gamma-glutamyltransferase (GGT) have been considered markers of oxidative stress. Some of our collaborators have already demonstrated that high serum GGT levels were an independent risk factor for all-cause mortality in PD patients by using the same cohort.^[50]^ Unfortunately, serum GGT levels were collected in only 836 (39.7%) patients of our cohort, so we could not include serum GGT levels as a covariate in our regression analysis. Furthermore, total bilirubin levels were not collected in this prospective cohort. Further translational research may be warranted to demonstrate the precise antioxidative effect of SUA on mortality.

In conclusion, our study demonstrated the impact of SUA on survival in ESRD patients. The TA-SUA <5.5 mg/dL is associated with a higher mortality in patients. This association was more prominent in aged, overweight, normoalbuminemic, well-nourished, and diabetic patients. The causal relationship is still obscure and further research should be warranted to clarify precise mechanism.

## Acknowledgments

The authors acknowledge the investigators of the cohort study (Clinical Research Center for End Stage Renal Disease) in South Korea.

## Supplementary Material

Supplemental Digital Content

## References

[1] Richette P Bardin T. Gout. *Lancet* 2010; 375:318–28. 19692116 10.1016/S0140-6736(09)60883-7

[2] Domrongkitchaiporn S Sritara P Kitiyakara C. Risk factors for development of decreased kidney function in a southeast Asian population: a 12-year cohort study. *J Am Soc Nephrol* 2005; 16:791–9. 15677313 10.1681/ASN.2004030208

[3] Obermayr RP Temml C Gutjahr G. Elevated uric acid increases the risk for kidney disease. *J Am Soc Nephrol* 2008; 19:2407–2413. 18799720 10.1681/ASN.2008010080 PMC2588108

[4] Weiner DE Tighiouart H Elsayed EF. Uric acid and incident kidney disease in the community. *J Am Soc Nephrol* 2008; 19:1204–1211. 18337481 10.1681/ASN.2007101075 PMC2396939

[5] Hsu CY Iribarren C McCulloch CE. Risk factors for end-stage renal disease: 25-year follow-up. *Arch Intern Med* 2009; 169:342–50. 19237717 10.1001/archinternmed.2008.605 PMC2727643

[6] Iseki K Ikemiya Y Inoue T. Significance of hyperuricemia as a risk factor for developing ESRD in a screened cohort. *Am J Kidney Dis* 2004; 44:642–50. 15384015

[7] Johnson RJ Kang DH Feig D. Is there a pathogenetic role for uric acid in hypertension and cardiovascular and renal disease?. *Hypertension* 2003; 41:1183–1190. 12707287 10.1161/01.HYP.0000069700.62727.C5

[8] Holme I Aastveit AH Hammar N. Uric acid and risk of myocardial infarction, stroke and congestive heart failure in 417,734 men and women in the Apolipoprotein MOrtality RISk study (AMORIS). *J Intern Med* 2009; 266:558–70. 19563390 10.1111/j.1365-2796.2009.02133.x

[9] Niskanen LK Laaksonen DE Nyyssonen K. Uric acid level as a risk factor for cardiovascular and all-cause mortality in middle-aged men: a prospective cohort study. *Arch Intern Med* 2004; 164:1546–1551. 15277287 10.1001/archinte.164.14.1546

[10] Fang J Alderman MH. Serum uric acid and cardiovascular mortality the NHANES I epidemiologic follow-up study, 1971-1992. National Health and Nutrition Examination Survey. *JAMA* 2000; 283:2404–2410. 10815083 10.1001/jama.283.18.2404

[11] Tsuruta Y Nitta K Akizawa T. Association between allopurinol and mortality among Japanese hemodialysis patients: results from the DOPPS. *Int Urol Nephrol* 2014; 46:1833–1841. 24908279 10.1007/s11255-014-0731-0 PMC4147244

[12] Rosolowsky ET Ficociello LH Maselli NJ. High-normal serum uric acid is associated with impaired glomerular filtration rate in nonproteinuric patients with type 1 diabetes. *Clin J Am Soc Nephrol* 2008; 3:706–13. 18272826 10.2215/CJN.04271007 PMC2386694

[13] Myllymaki J Honkanen T Syrjanen J. Uric acid correlates with the severity of histopathological parameters in IgA nephropathy. *Nephrol Dial Transplant* 2005; 20:89–95. 15572382 10.1093/ndt/gfh584

[14] Syrjanen J Mustonen J Pasternack A. Hypertriglyceridaemia and hyperuricaemia are risk factors for progression of IgA nephropathy. *Nephrol Dial Transplant* 2000; 15:34–42. 10.1093/ndt/15.1.34 10607765

[15] Madero M Sarnak MJ Wang X. Uric acid and long-term outcomes in CKD. *Am J Kidney Dis* 2009; 53:796–803. 19303683 10.1053/j.ajkd.2008.12.021 PMC2691553

[16] Suliman ME Johnson RJ Garcia-Lopez E. J-shaped mortality relationship for uric acid in CKD. *Am J Kidney Dis* 2006; 48:761–71. 17059995 10.1053/j.ajkd.2006.08.019

[17] Liu WC Hung CC Chen SC. Association of hyperuricemia with renal outcomes, cardiovascular disease, and mortality. *Clin J Am Soc Nephrol* 2012; 7:541–8. 22300737 10.2215/CJN.09420911

[18] Navaneethan SD Beddhu S. Associations of serum uric acid with cardiovascular events and mortality in moderate chronic kidney disease. *Nephrol Dial Transplant* 2009; 24:1260–6. 19033255 10.1093/ndt/gfn621 PMC2721426

[19] Hsu SP. Serum uric acid levels show a ’J-shaped’ association with all-cause mortality in haemodialysis patients. *Nephrol Dial Transplant* 2004; 19:457–62. 14736974 10.1093/ndt/gfg563

[20] Lee SM Lee AL Winters TJ. Low serum uric acid level is a risk factor for death in incident hemodialysis patients. *Am J Nephrol* 2009; 29:79–85. 18689987 10.1159/000151292 PMC2786018

[21] Latif W Karaboyas A Tong L. Uric acid levels and all-cause and cardiovascular mortality in the hemodialysis population. *Clin J Am Soc Nephrol* 2011; 6:2470–7. 21868616 10.2215/CJN.00670111 PMC3359562

[22] Chen Y Ding X Teng J. Serum uric acid is inversely related to acute ischemic stroke morbidity in hemodialysis patients. *Am J Nephrol* 2011; 33:97–104. 21196722 10.1159/000322966

[23] Park JT Kim DK Chang TI. Uric acid is associated with the rate of residual renal function decline in peritoneal dialysis patients. *Nephrol Dial Transplant* 2009; 24:3520–5. 19491381 10.1093/ndt/gfp272

[24] Tang Z Cheng LT Li HY Wang T. Serum uric acid and endothelial dysfunction in continuous ambulatory peritoneal dialysis patients. *Am J Nephrol* 2009; 29:368–73. 18974637 10.1159/000168484

[25] Feng S Jiang L Shi Y. Uric acid levels and all-cause mortality in peritoneal dialysis patients. *Kidney Blood Press Res* 2013; 37:181–9. 23736777 10.1159/000350143

[26] Xia X He F Wu X. Relationship between serum uric acid and all-cause and cardiovascular mortality in patients treated with peritoneal dialysis. *Am J Kidney Dis* 2014; 64:257–64. 24176223 10.1053/j.ajkd.2013.08.027

[27] Glantzounis GK Tsimoyiannis EC Kappas AM Galaris DA. Uric acid and oxidative stress. *Curr Pharm Des* 2005; 11:4145–4151. 16375736 10.2174/138161205774913255

[28] Ames BN Cathcart R Schwiers E Hochstein P. Uric acid provides an antioxidant defense in humans against oxidant- and radical-caused aging and cancer: a hypothesis. *Proc Natl Acad Sci U S A* 1981; 78:6858–6862. 6947260 10.1073/pnas.78.11.6858 PMC349151

[29] Maxwell SR Thomason H Sandler D. Antioxidant status in patients with uncomplicated insulin-dependent and non-insulin-dependent diabetes mellitus. *Eur J Clin Invest* 1997; 27:484–90. 9229228 10.1046/j.1365-2362.1997.1390687.x

[30] Choi JY Jang HM Park J. Survival advantage of peritoneal dialysis relative to hemodialysis in the early period of incident dialysis patients: a nationwide prospective propensity-matched study in Korea. *PLoS One* 2013; 8:e84257. 24386357 10.1371/journal.pone.0084257 PMC3875495

[31] Lee J An JN Hwang JH. Effect of dialysis initiation timing on clinical outcomes: a propensity-matched analysis of a prospective cohort study in Korea. *PLoS One* 2014; 9:e105532. 25137235 10.1371/journal.pone.0105532 PMC4138196

[32] http://www.kosis.kr/.

[33] Chen JH Yeh WT Chuang SY. Gender-specific risk factors for incident gout: a prospective cohort study. *Clin Rheumatol* 2012; 31:239–45. 21761146 10.1007/s10067-011-1802-6

[34] Beberashvili I Sinuani I Azar A. Serum uric acid as a clinically useful nutritional marker and predictor of outcome in maintenance hemodialysis patients. *Nutrition* 2015; 31:138–47. 25466658 10.1016/j.nut.2014.06.012

[35] Gephardt MC Hanlon TJ Matson CF. Blood uric acid values as related to sex and age. *JAMA* 1964; 189:1028–9. 14188883 10.1001/jama.1964.03070130048019

[36] Hak AE Choi HK. Menopause, postmenopausal hormone use and serum uric acid levels in US women—the Third National Health and Nutrition Examination Survey. *Arthritis Res Ther* 2008; 10:R116. 18822120 10.1186/ar2519 PMC2592803

[37] Palmer BF. Sexual dysfunction in uremia. *J Am Soc Nephrol* 1999; 10:1381–8. 10361878 10.1681/ASN.V1061381

[38] Gouri A Dekaken A Bentorki AA. Serum uric acid level and cardiovascular risks in hemodialysis patients: an Algerian cohort study. *Clin Lab* 2014; 60:751–8. 24839817 10.7754/clin.lab.2013.130310

[39] Dong J Han QF Zhu TY. The associations of uric acid, cardiovascular and all-cause mortality in peritoneal dialysis patients. *PLoS One* 2014; 9:e82342. 24416142 10.1371/journal.pone.0082342 PMC3885378

[40] Vashistha T Mehrotra R Park J. Effect of age and dialysis vintage on obesity paradox in long-term hemodialysis patients. *Am J Kidney Dis* 2014; 63:612–22. 24120224 10.1053/j.ajkd.2013.07.021 PMC3969454

[41] Vincent JL Dubois MJ Navickis RJ Wilkes MM. Hypoalbuminemia in acute illness: is there a rationale for intervention? A meta-analysis of cohort studies and controlled trials. *Ann Surg* 2003; 237:319–34. 12616115 10.1097/01.SLA.0000055547.93484.87 PMC1514323

[42] Detsky AS McLaughlin JR Baker JP. What is subjective global assessment of nutritional status?. *JPEN J Parenter Enteral Nutr* 1987; 11:8–13. 3820522 10.1177/014860718701100108

[43] Kwon YE Kee YK Yoon CY. Change of nutritional status assessed using subjective global assessment is associated with all-cause mortality in incident dialysis patients. *Medicine (Baltimore* 2016; 95:e2714. 26886609 10.1097/MD.0000000000002714 PMC4998609

[44] Alvarez-Lario B Macarron-Vicente J. Uric acid and evolution. *Rheumatology* 2010; 49:2010–5. 20627967 10.1093/rheumatology/keq204

[45] Waring WS McKnight JA Webb DJ Maxwell SR. Uric acid restores endothelial function in patients with type 1 diabetes and regular smokers. *Diabetes* 2006; 55:3127–3132. 17065352 10.2337/db06-0283

[46] Waring WS Convery A Mishra V. Uric acid reduces exercise-induced oxidative stress in healthy adults. *Clin Sci* 2003; 105:425–30. 10.1042/CS20030149 12801243

[47] Andreadou E Nikolaou C Gournaras F. Serum uric acid levels in patients with Parkinson's disease: their relationship to treatment and disease duration. *Clin Neurol Neurosurg* 2009; 111:724–8. 19632030 10.1016/j.clineuro.2009.06.012

[48] Wu VC Huang JW Hsueh PR. Renal hypouricemia is an ominous sign in patients with severe acute respiratory syndrome. *Am J Kidney Dis* 2005; 45:88–95. 15696447 10.1053/j.ajkd.2004.09.031 PMC7115701

[49] Kim SB Yang WS Min WK. Reduced oxidative stress in hypoalbuminemic CAPD patients. *Perit Dial Int* 2000; 20:290–4. 10898045

[50] Park WY Kim SH Kim YO. Serum gamma-glutamyltransferase levels predict mortality in patients with peritoneal dialysis. *Medicine (Baltimore* 2015; 94:e1249. 26252286 10.1097/MD.0000000000001249 PMC4616583

