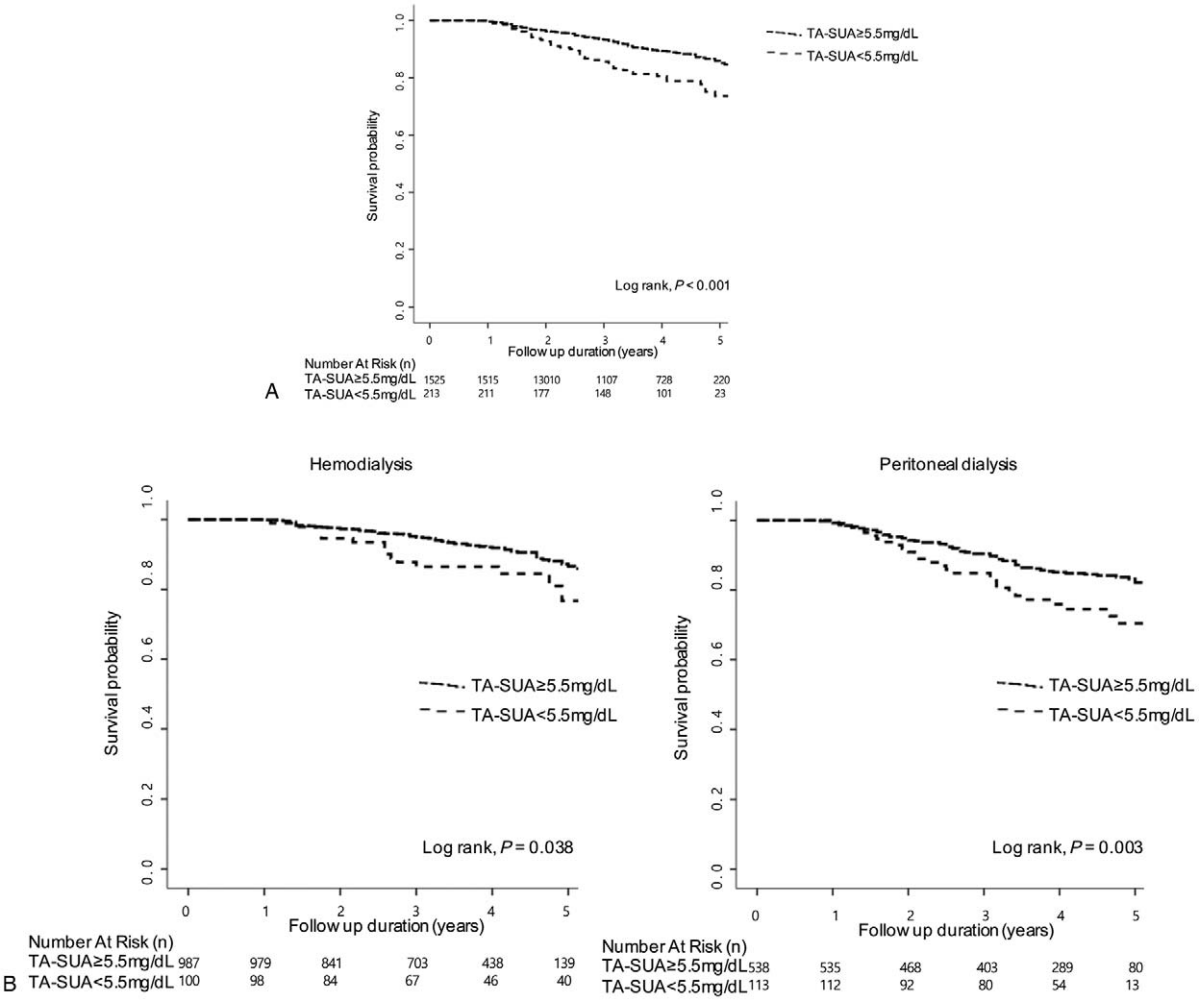# Lower serum uric acid level predicts mortality in dialysis patients: Erratum

**DOI:** 10.1097/MD.0000000000027274

**Published:** 2021-09-17

In the article, “Lower serum uric acid level predicts mortality in dialysis patients”,^[1]^ which appears in Volume 95, Issue 24 of *Medicine*, Figure 3 was repeated both as Figure 3 and 4. Figure 4 has now been corrected to the below figure:

**Figure:**