# Supplementary material for: Lower serum uric acid level predicts mortality in dialysis patients
Source: Medicine (Baltimore). 2016 Jun 14;95(24):e3701. doi: 10.1097/MD.0000000000003701 (PMC4998435; doi:10.1097/MD.0000000000003701)
Supplement: Supplemental Digital Content [file medi-95-e3701-s001.doc]

Supplementary Table 1. Patient characteristics by the time averaged-serum uric acid groups in hemodialysis group

| **Variables** | **Total**  **(N = 1,087)** | **< 5.5**  **(N = 100)** | **5.5–6.4**  **(N = 218)** | **6.5–7.4**  **(N = 317)** | **7.5–8.4**  **(N = 256)** | **≥ 8.5**  **(N = 196)** | ***P*** | ***N (%)*** |
| --- | --- | --- | --- | --- | --- | --- | --- | --- |
| Age (years) | 56 ± 13 | 59 ± 15 | 60 ± 13 | 58 ± 13 | 58 ± 12 | 54 ± 14 | 0.012 | 1087 (100) |
| Men (N, %) | 604(55.6) | 51 (51.0) | 108 (49.5) | 179 (56.5) | 143 (55.9) | 123 (62.9) | 0.082 | 1087 (100) |
| BMI (kg/m^2^) | 22.3 ± 3.3 | 20.9 ± 2.9 | 21.9 ± 3.0 | 22.4 ± 3.4 | 22.5 ± 3.4 | 23.0 ± 3.2 | <0.001 | 1073 (98.7) |
| SBP (mmHg) | 140 ± 21 | 142 ± 22 | 142 ± 20 | 140 ± 19 | 140 ± 21 | 140 ± 22 | 0.678 | 1053 (96.9) |
| DBP (mmHg) | 76 ± 13 | 76 ± 13 | 76 ± 13 | 75 ± 12 | 76 ± 13 | 80 ± 12 | 0.003 | 1050 (99.7) |
| Hb (g/dL) | 10.7 ± 1.2 | 10.7 ± 1.1 | 10.6 ± 1.3 | 10.8 ± 1.2 | 1075 ± 1.2 | 10.8 ± 1.2 | 0.543 | 1084 (99.7) |
| HbA1c (%) | 6.5 ± 1.5 | 7.0 ± 1.5 | 6.4 ± 1.5 | 6.6 ± 1.5 | 6.4 ± 1.5 | 6.3 ± 1.4 | 0.034 | 642 (59.1) |
| Ca (mg/dL) | 8.8 ± 0.9 | 9.0 ± 0.9 | 8.8 ± 1.0 | 8.8 ± 0.9 | 8.8 ± 0.8 | 8.8 ± 0.9 | 0.125 | 1080 (99.4) |
| P (mg/dL) | 5.0 ± 1.5 | 4.3 ± 1.4 | 4.7 ± 1.5 | 5.0 ± 1.3 | 4.9 ± 1.4 | 5.4 ± 1.6 | <0.001 | 1081 (99.4) |
| Total Protein (g/dL) | 6.8 ± 0.6 | 6.9 ± 0.6 | 6.8 ± 0.7 | 6.8 ± 0.6 | 6.8 ± 0.5 | 6.9 ± 0.5 | 0.005 | 1080 (99.4) |
| Albumin (g/dL) | 3.9 ± 0.4 | 4.0 ± 0.5 | 3.9 ± 0.4 | 3.9 ± 0.4 | 3.9 ± 0.3 | 4.0 ± 0.3 | 0.003 | 1082 (99.5) |
| Chol (mg/dL) | 153 ± 36 | 151 ± 37 | 154 ± 38 | 152 ± 36 | 152 ± 36 | 155 ± 35 | 0.876 | 1059 (97.4) |
| TG (mg/dL) | 121 ± 93 | 102 ± 73 | 126 ± 148 | 114 ± 61 | 114 ± 69 | 143 ± 86 | 0.012 | 999 (91.9) |
| LDL (mg/dL) | 83 ± 29 | 83 ± 28 | 83 ± 30 | 83 ± 29 | 82 ± 30 | 82 ± 29 | 0.998 | 891 (82.0) |
| HDL (mg/dL) | 42 ± 13 | 44 ± 14 | 43 ± 14 | 41 ± 13 | 42 ± 14 | 41 ± 12 | 0.173 | 985 (90.6) |
| hs-CRP (mg/dL) | 1.7 ± 8.8 | 2.1 ± 10.7 | 1.9 ± 6.6 | 1.8 ± 10.0 | 2.0 ± 10.7 | 0.7 ± 3.0 | 0.643 | 938 (86.3) |
| SGA (malnourished, %) | 131 (13.0) | 18 (18.2) | 27 (13.2) | 46 (15.7) | 26 (10.9) | 14 (8.0) | 0.057 | 1011(93.0) |
| Total Kt/V (per week) | 1.51 ± 0.41 | 1.62 ± 0.52 | 1.55 ± 0.68 | 1.50 ± 0.30 | 1.51 ± 0.28 | 1.51 ± 0.41 | 0.051 | 972 (89.4) |
| Dialysis duration (month) | 35 (16 – 72) | 35 (19 - 78) | 37 (16 - 80) | 33 (14 - 70) | 36 (15 - 69) | 36 (15 - 72) | 0.742 | 1073 (98.7) |
| Follow up duration (month) | 42.9 ± 14.6 | 43.1 ± 14.8 | 41.8 ± 14.6 | 42.6 ± 15.3 | 43.5 ± 14.0 | 43.6 ± 14.0 | 0.689 | 1087 (100) |
| Etiology of ESRD |  |  |  |  |  |  | 0.005 |  |
| DM (N, %) | 455 (50.4) | 52 (60.5) | 89 (48.4) | 157 (59.7) | 92 (43.6) | 65 (41.1) |  | 455 (50.4) |
| HTN (N, %) | 202 (22.4) | 11 (12.8) | 42 (22.8) | 50 (19.0) | 53 (25.1) | 46 (29.1) |  | 202 (22.4) |
| GN (N, %) | 146 (16.2) | 14 (16.3) | 29 (15.8) | 27 (10.3) | 42 (19.9) | 34 (21.5) |  | 146 (16.2) |
| Cystic disease | 54 (6.0) | 4 (4.6) | 13 (7.1) | 16 (6.1) | 15(7.1) | 6 (3.8) |  | 54 (6.0) |
| Miscellaneous condition | 45 (5.0) | 5 (5.8) | 11 (6.0) | 13 (5.0) | 9 (4.2) | 7 (4.4) |  | 45 (5.0) |
| Comorbid conditions |  |  |  |  |  |  |  |  |
| DM (N, %) | 458 (53.4) | 51 (61.4) | 89 (50.9) | 157 (59.0) | 94 (49.0) | 67 (47.2) | 0.045 | 458 (53.4) |
| CAD (N, %) | 80 (10.3) | 7 (9.1) | 12 (7.6) | 27 (11.4) | 16 (9.0) | 18 (14.4) | 0.377 | 80 (10.3) |
| PVD (N, %) | 11 (1.4) | 1 (1.3) | 2 (1.3) | 3 (1.3) | 3 (1.7) | 2 (1.6) | 0.996 | 11 (1.4) |
| CVD (N, %) | 40 (5.2) | 1 (1.3) | 11 (7.0) | 11 (4.6) | 5 (2.8) | 12 (9.6) | 0.031 | 40 (5.2) |
| CHF (N, %) | 48 (6.2) | 4 (5.2) | 11 (7.0) | 19 (8.0) | 7 (3.9) | 7 (5.6) | 0.508 | 48 (6.2) |
| Gout (N, %) | 51 (4.7) | 3 (3.0) | 12 (5.5) | 14 (4.4) | 11 (4.3) | 11 (5.6) | 0.831 | 51 (4.7) |
| Tumor (N, %) | 29 (3.7) | 3 (3.9) | 7 (4.5) | 7 (3.0) | 5 (2.8) | 7 (5.6) | 0.687 | 29 (3.7) |
| Uricosuric drugs (N, %) | 600 (55.2) | 61 (61.0) | 129 (59.2) | 177 (55.8) | 136 (53.1) | 97 (49.5) | 0.212 | 600 (55.2) |
| Antiuricosuric drugs (N, %) | 931 (85.6) | 77 (77.0) | 188 (86.2) | 269 (84.9) | 220 (85.9) | 177 (90.3) | 0.044 | 931 (85.6) |

BMI, body mass index; SBP, systolic blood pressure; DBP, diastolic blood pressure; Hb, hemoglobin; Ca, calcium; P, phosphorus; Chol, cholesterol; TG, triglycerides; LDL, low-density lipoprotein; HDL, high-density lipoprotein; hs-CRP, high-sensitivity C-reactive protein; SGA, subjective global assessment; ESRD, end stage renal disease; DM, diabetes; GN, glomerular nephritis; CAD, coronary artery disease; PVD, peripheral vascular disease; CVD, cerebrovascular disease; CHF, congestive heart failure

Supplementary Table 2. Patient characteristics by the time averaged-serum uric acid groups in peritoneal dialysis group

| **Variables** | **Total**  **(N = 651)** | **< 5.5**  **(N = 113)** | **5.5–6.4**  **(N = 182)** | **6.5–7.4**  **(N = 176)** | **7.5–8.4**  **(N = 115)** | **≥ 8.5**  **(N = 65)** | ***P*** | ***N (%)*** |
| --- | --- | --- | --- | --- | --- | --- | --- | --- |
| Age (years) | 53 ± 12 | 56 ± 12 | 54 ± 12 | 52 ± 12 | 51 ± 13 | 52 ± 12 | 0.010 | 651 (100) |
| Men (N, %) | 348 (53.5) | 51 (45.1) | 84 (46.2) | 98 (55.7) | 73 (63.5) | 42 (64.6) | 0.004 | 651 (100) |
| BMI (kg/m^2^) | 23.7 ± 3.1 | 23.4 ± 3.4 | 23.3 ± 2.9 | 23.7 ± 2.7 | 23.8 ± 3.3 | 24.8 ± 3.2 | 0.022 | 579 (88.9) |
| SBP (mmHg) | 133 ± 21 | 128 ± 24 | 134 ± 20 | 133 ± 22 | 135 ± 17 | 132 ± 21 | 0.117 | 584 (89.7) |
| DBP (mmHg) | 79 ± 12 | 77 ± 11 | 80 ± 11 | 80 ± 12 | 80 ± 12 | 80 ± 14 | 0.141 | 583 (89.6) |
| Hb (g/dL) | 10.5 ± 1.5 | 10.6 ± 1.5 | 10.3 ± 1.4 | 10.8 ± 1.5 | 10.2 ± 1.4 | 10.5 ± 1.4 | 0.002 | 648 (99.5) |
| HbA1c (%) | 6.4 ± 2.2 | 6.9 ± 1.8 | 6.5 ± 2.8 | 6.2 ± 1.3 | 6.1 ± 1.0 | 6.6 ± 3.7 | 0.311 | 307 (47.2) |
| Ca (mg/dL) | 8.7 ± 0.9 | 8.7 ± 0.8 | 8.7 ± 0.9 | 8.9 ± 0.8 | 8.7 ± 0.8 | 8.6 ± 0.9 | 0.126 | 637 (97.8) |
| P (mg/dL) | 5.1 ± 1.5 | 4.5 ± 1.4 | 4.9 ± 1.3 | 5.1 ± 1.5 | 5.5 ± 1.5 | 5.7 ± 1.7 | <0.001 | 637 (97.8) |
| Total Protein (g/dL) | 6.5 ± 0.7 | 6.4 ± 0.6 | 6.4 ± 0.7 | 6.5 ± 0.6 | 6.5 ± 0.6 | 6.7 ± 0.8 | 0.028 | 630 (96.7) |
| Albumin (g/dL) | 3.7 ± 0.5 | 3.5 ± 0.4 | 3.6 ± 0.5 | 3.7 ± 0.4 | 3.7 ± 0.4 | 3.8 ± 0.6 | 0.001 | 628 (96.4) |
| Chol (mg/dL) | 177 ± 40 | 177 ± 40 | 176 ± 40 | 177 ± 40 | 179 ± 39 | 175 ± 38 | 0.971 | 604 (92.8) |
| TG (mg/dL) | 154 ± 101 | 144 ± 85 | 150 ± 97 | 152 ± 105 | 163 ± 95 | 177 ± 130 | 0.401 | 469 (72.2) |
| LDL (mg/dL) | 105 ± 33 | 110 ± 28 | 105 ± 31 | 103 ± 35 | 106 ± 33 | 104 ± 39 | 0.742 | 470 (74.6) |
| HDL (mg/dL) | 41 ± 13 | 42 ± 12 | 42 ± 13 | 42 ± 13 | 38 ± 12 | 39 ± 9 | 0.143 | 406 (62.4) |
| hs-CRP (mg/dL) | 0.5 ± 1.9 | 0.7 ± 1.8 | 0.3 ± 0.9 | 0.7 ± 2.9 | 0.5 ± 1.9 | 0.4 ± 1.2 | 0.567 | 401 (61.6) |
| SGA (malnourished, %) | 76 (14.1) | 14 (14.9) | 24 (15.8) | 23 (15.9) | 11 (11.5) | 4 (7.5) | 0.522 | 540 (82.9) |
| Total Kt/V (per week) | 1.68 ± 0.54 | 1.68 ± 0.52 | 1.84 ± 0.53 | 1.71 ± 0.57 | 1.47 ± 0.43 | 1.47 ± 0.49 | <0.001 | 490 (75.3) |
| Dialysis duration (month) | 39 (16 – 67) | 44 (15 - 81) | 42 (19 - 72) | 39 (18 - 66) | 42 (18 - 57) | 21 (13 - 47) | 0.007 | 627 (96.3) |
| Follow up duration (month) | 45.5 ± 14.8 | 43.8 ± 15.1 | 45.9 ± 15.4 | 45.2 ± 14.7 | 46.0 ± 13.7 | 45.5 ± 14.8 | 0.575 | 651 (100) |
| Etiology of ESRD |  |  |  |  |  |  | 0.124 |  |
| DM (N, %) | 194 (38.6) | 42 (51.9) | 46 (35.1) | 52 (36.6) | 33 (35.1) | 21 (38.9) |  | 194 (38.6) |
| HTN (N, %) | 155 (30.9) | 24 (29.6) | 44 (33.6) | 45 (31.7) | 21 (22.3) | 21 (38.9) |  | 155 (30.9) |
| GN (N, %) | 121 (24.1) | 12 (14.8) | 34 (26.0) | 32 (22.5) | 33 (35.1) | 10 (18.5) |  | 121 (24.1) |
| Cystic disease | 21 (4.2) | 1 (1.2) | 4 (3.1) | 10 (7.0) | 5 (5.3) | 1 (1.9) |  | 21 (4.2) |
| Miscellaneous condition | 11 (2.2) | 2 (2.4) | 3 (2.3) | 3 (2.1) | 2 (2.1) | 1 (1.9) |  | 11 (2.2) |
| Comorbid conditions |  |  |  |  |  |  |  |  |
| DM (N, %) | 309 (57.3) | 50 (50.0) | 68 (43.6) | 55 (38.5) | 35 (39.3) | 22 (43.1) | 0.449 | 309 (57.3) |
| CAD (N, %) | 45 (8.8) | 10 (10.5) | 16 (10.8) | 11 (8.0) | 3 (3.7) | 5 (10.6) | 0.404 | 45 (8.8) |
| PVD (N, %) | 17 (3.3) | 4 (4.2) | 4 (2.7) | 5 (3.6) | 2 (2.5) | 2 (4.3) | 0.944 | 17 (3.3) |
| CVD (N, %) | 26 (5.1) | 5 (5.3) | 7 (4.7) | 7 (5.1) | 1 (1.2) | 6 (12.8) | 0.083 | 26 (5.1) |
| CHF (N, %) | 42 (8.3) | 10 (10.5) | 8 (5.4) | 11 (8.0) | 5 (6.2) | 8 (17.0) | 0.112 | 42 (8.3) |
| Gout (N, %) | 29 (4.5) | 5 (4.4) | 7 (3.8) | 10 (5.7) | 2 (1.7) | 5 (7.7) | 0.358 | 29 (4.5) |
| Tumor (N, %) | 11 (2.2) | 2 (2.1) | 3 (2.0) | 5 (3.6) | 1 (1.2) | 0 (0.0) | 0.596 | 11 (2.2) |
| Uricosuric drugs (N, %) | 406 (62.4) | 72 (63.7) | 110 (60.4) | 110 (62.5) | 79 (68.7) | 35 (53.8) | 0.495 | 578 (88.8) |
| Antiuricosuric drugs (N, %) | 578 (88.8) | 99 (87.6) | 158 (86.8) | 162 (92.0) | 100 (87.0) | 59 (90.8) | 0.495 | 578 (88.8) |

BMI, body mass index; SBP, systolic blood pressure; DBP, diastolic blood pressure; Hb, hemoglobin; Ca, calcium; P, phosphorus; Chol, cholesterol; TG, triglycerides; LDL, low-density lipoprotein; HDL, high-density lipoprotein; hs-CRP, high-sensitivity C-reactive protein; SGA, subjective global assessment; ESRD, end stage renal disease; DM, diabetes; GN, glomerular nephritis; CAD, coronary artery disease; PVD, peripheral vascular disease; CVD, cerebrovascular disease; CHF, congestive heart failure
